# Measuring the disinfection efficacy of the Tru-D iQ and Tru-D Legacy systems in acute patient care hospital rooms: a randomized experimental study

**DOI:** 10.1017/ash.2025.10223

**Published:** 2025-11-17

**Authors:** Bobby G. Warren, Guerbine Fils-Aime, Amanda M. Graves, Aaron Barrett, Nicholas A. Turner, Deverick J. Anderson

**Affiliations:** 1 Duke Center for Antimicrobial Stewardship and Infection Prevention, Durham, NC, USA; 2 Disinfection Resistance and Transmission Epidemiology (DiRTE) Lab, Duke University School of Medicine, Durham, NC, USA; 3 Division of Infectious Diseases, Duke University Medical Center, Durham, NC, USA

## Abstract

In this randomized experimental study, Tru-D iQ and Legacy UV-C systems both reduced environmental contamination in hospital rooms. Both systems significantly reduced contamination, with the Tru-D iQ achieving comparable efficacy overall and significantly shorter cycle times.

## Introduction

Over 700,000 healthcare-associated infections (HAIs) and 99,000 related deaths occur annually in the United States.^1,2^ The healthcare environment is a key contributor to HAI transmission.^3–5^ Therefore, effective environmental disinfection is critical, yet standard chemical disinfection is limited by compliance and contact time; thus, enhanced disinfection strategies are needed.^6–8^

Previously, the Tru-D UV-C system (PDI Healthcare) demonstrated effectiveness in decreasing the incidence of MRSA and VRE in high-risk hospital rooms and hospitalwide incidence of *Clostridioides difficile* and VRE when used for terminal disinfection without concurrent chlorine-based disinfectants.^9,10^ Bathrooms received only indirect UV-C, and run times often exceeded 45 minutes, limiting feasibility.

An improved UV-C delivery system (Tru-D iQ) was developed to improve direct UV-C coverage and reduced disinfection run times. We completed a randomized experimental study to assess whether the Tru-D iQ demonstrated superior disinfection efficacy and operational performance compared with the Tru-D Legacy system after patient discharge.

## Methods

### Study setting and design

We performed this study in inpatient rooms at Duke University Hospital, a 1 048-bed tertiary care hospital in Durham, North Carolina.

Rooms were randomized 1:1 to intervention (Tru-D iQ) or control (Tru-D Legacy). Randomization was stratified by room size so that once a room of a particular size category was assigned to one device, the next room of that size was assigned to the alternate device to maintain size balance across arms. Both Tru-D iQ and Tru-D Legacy systems use real-time sensors to end cycles once the target fluence is reached, with duration adjusting automatically to room size and reflectivity. Each system employs preset cycles specific to vegetative or spore-forming bacteria. The iQ includes a compact secondary emitter for direct bathroom exposure, while the Legacy uses a single-unit emitter for the main room only. Clinical, laboratory, and statistical staff were blinded to randomization; device operators were not.

Six areas in each study room, three in the main room (bed rails, overbed table, and in-room sink) and three in the patient bathroom (toilet seat, bathroom floor around the base of the toilet, and the bathroom sink) were split in half by left and right sides and randomized to pre and postdisinfection sampling.

Additionally, 10 x 10 cm formica sheets were inoculated with 1 x 10^4^ CFU of USA300 Methicillin-resistant *Staphylococcus aureus* (vegetative disinfection cycles) or Ribotype 027 *C. difficile* (spore cycles) and placed in three predetermined locations: medical prep area, computer keyboard, and toilet seat.

### Study protocol

Rooms were enrolled immediately following the discharge of a patient on contact precautions and prior to any physical cleaning or chemical disinfection. Each room was randomized to the intervention or control study arm, and sample areas were further randomized by pre and postdisinfection sampling as described above. Any item obstructing sampling, such as linens, were removed. The disinfection cycle was selected according to the pathogen that had required contact precautions for the discharged patient. Inoculated formica sheets were placed at predetermined locations, and the assigned UV-C system was used to disinfect the room. Following disinfection, samples were collected from postdisinfection surfaces and formica sheets.

### Microbiological methods

All environmental cultures were obtained using the sponge–stomacher technique as previously described, with homogenates plated onto routine and selective media for clinically important pathogens (CIPs), defined as *S. aureus*, *Enterococcus spp.*, Enterobacterales, and *C. difficile*. Species were confirmed via 16S rRNA sequencing.^5,8^

### Outcomes

The primary outcome was the percent reduction in total contamination, measured in colony-forming units (CFUs), from pre to postdisinfection on environmental surfaces. Results from the intervention arm were compared against results from the control arm. Total room CFUs were determined separately for pre and postdisinfection samples by summing the CFUs from all six sampled surfaces on a given side (eg, all right-side surfaces or all left-side surfaces). For analysis, these six sites together were defined as the “room–bathroom suite,“ while the three main room sites (bedrails, overbed table, and room sink) were grouped as “room“ and the three-bathroom sites (toilet seat, bathroom floor, and bathroom sink) were grouped as “bathroom.“ Secondary outcomes were the percent reduction in CFU counts for CIPs, percent reduction in surfaces positive for CIPs, elimination of inoculated pathogens on formica sheets, and device run time.

### Statistical analysis

The Wilcoxon signed-rank test was used to compare overall and CIP CFU percent reduction between study arms, device run time, and the Fisher’s exact test was used to compare proportions of sample areas with CIPs and pathogen elimination on formica sheets. *P* < .05 was considered to be significant. All statistical tests were two-tailed and performed using SAS, version 9.4M7 (SAS Institute Inc). This study was reviewed by the Duke University Health System Institutional Review Board and received an “exempt” status.

## Results

Thirty rooms were enrolled: 16 intervention and 14 control. Of those, 18 received the vegetative cycle (10 intervention, 8 control), and 12 received the spore cycle (6 per group). Overall, 396 samples were collected. In general, predisinfection CFU counts were substantial: 23,845 (IQR 1,958–75,360) in suites, with the bathroom (13,407; IQR 1,353–57,538) and bathroom floor (11,701; IQR 1,079–45,225) most contaminated.

Overall, both arms showed bioburden reduction at the suite, room, bathroom, and surface levels (Table 1). Median percent reduction was generally greater in rooms treated with the Tru-D iQ system compared to the Tru-D Legacy system, though most differences were not statistically significant. Notably, bathroom sinks demonstrated a significantly greater reduction with iQ [83% (IQR 18–100)] compared to Legacy [68% (IQR 30–84); *P* = .02]. For CIPs, both arms showed reductions, with iQ often greater but not significant. Species-level analysis reported as proportion of positive samples demonstrated variable reductions, with no consistent differences between devices (Table 2).

**Table 1.** Median (IQR) CFU counts before and after UV-C disinfection by surface type and device

	Control arm			Intervention arm			
	Before	After	Percent reduction	Before	After	Percent reduction	*p*
**All CFU (Median [IQR])**							
Room-Bathroom suite	56,096 (17,592–112,182)	10,150 (2,430–74,314)	48 (23–88)	4,255 (1,708–68,319)	2,262 (693–21,655)	35 (17–82)	.37
Patient room	6,288 (349–12,123)	910 (35–2,280)	85 (66–95)	1,422 (176–6,894)	180 (0–1,454)	93 (70–100)	.74
Bedrails	1,890 (368–8,690)	234 (0–822)	95 (67–100)	150 (0–1,550)	0 (0–506)	99 (67–100)	.18
Overbed table	2,646 (720–6,700)	49 (0–1,325)	91 (62–100)	141 (0–1,125)	0 (0–180)	100 (96–100)	.82
Room sink	1,426 (221–3,453)	98 (0–915)	98 (89–100)	228 (0–1,462)	15 (0–166)	99 (85–100)	.56
Patient bathroom	38,984 (15,055–101,426)	9,013 (2,390–70,583)	68 (27–88)	3,425 (1,214–20,820)	1,300 (288–8,344)	79 (13–89)	.8
Toilet seat	3,970 (81–15,818)	708 (0–11,822)	75 (58–93)	73 (0–1,094)	0 (0–578)	100 (70–100)	.17
Bathroom floor	34,205 (15,970–53,079)	7,318 (2,632–56,232)	70 (21–88)	1,954 (476–6,659)	805 (13–6,297)	71 (95–100)	.94
Bathroom sink	2,420 (980–5,764)	1,222 (110–2,490)	68 (30–84)	500 (150–901)	135 (0–700)	83 (18–100)	.02
**CIP CFU (Median [IQR])**							
Room-Bathroom suite	7,689 (2,898–16,925)	3,379 (723–8,653)	46 (27–63)	954 (501–2,352)	194 (18–1,434)	60 (38–97)	.22
Patient room	3,923 (490–4,888)	88 (0–349)	98 (45–100)	130 (0–964)	0 (0–25)	87 (0–100)	.86
Bedrails	622 (13–3,162)	0 (0–0)	99 (0–100)	0 (0–12)	0 (0–0)	0 (0–47)	.73
Overbed table	382 (0–2,153)	0 (0–41)	55 (0–100)	0 (0–52)	0 (0–0)	0 (0–66)	.13
Room sink	98 (0–896)	12 (0–169)	53 (0–99)	0 (0–215)	0 (0–0)	0 (0–100)	1.0
Patient bathroom	3,719 (1,118–12,420)	2,654 (483–8,079)	24 (10–72)	766 (68–1,377)	167 (18–1,248)	22 (0–86)	.88
Toilet seat	0 (0–915)	0 (0–117)	0 (0–69)	0 (0–51)	0 (0–265)	0 (0–59)	.83
Bathroom floor	2,270 (448–7,471)	768 (39–6,229)	32 (60–89)	286 (0–908)	54 (0–495)	28 (0–85)	.57
Bathroom sink	786 (198–2,489)	132 (0–1,212)	59 (12–100)	0 (0–147)	0 (0–37)	0 (0–42)	.14

**Table 2.** Proportion of surfaces positive for clinically important pathogens before and after UV-C disinfection by surface type, device, and species

	Control arm		Intervention arm		
	Before *N* (%)	After *N* (%)	Before *N* (%)	After *N* (%)	*p*
**Overall, Presence of any CIP**					
Room-Bathroom suite	61 (73)	41 (49)	45 (48)	32 (34)	.83
Patient Room	27 (64)	15 (36)	20 (43)	9 (19)	.56
Bedrails	10 (71)	3 (21)	4 (27)	2 (13)	.58
Overbed table	8 (57)	5 (36)	8 (50)	3 (19)	.62
Room sink	9 (64)	7 (50)	8 (50)	4 (25)	.34
Patient bathroom	34 (81)	26 (62)	25 (53)	23 (49)	.17
Toilet seat	7 (50)	5 (36)	6 (38)	7 (44)	.46
Bathroom floor	14 (100)	12 (86)	12 (75)	11 (69)	1.00
Bathroom sink	13 (93)	9 (64)	7 (47)	5 (33)	1.00
***S. aureus***					
Room-Bathroom suite	40 (48)	21 (25)	17 (18)	8 (9)	.78
Patient room	20 (48)	4 (10)	7 (15)	4 (9)	.15
Bedrails	6 (43)	1 (7)	3 (20)	1 (7)	1.00
Overbed table	8 (57)	2 (14)	3 (19)	3 (19)	.06
Room sink	6 (43)	1 (7)	1 (6)	0 (0)	1.00
Patient bathroom	20 (48)	17 (40)	10 (21)	4 (9)	.03
Toilet seat	4 (29)	4 (29)	4 (25)	1 (6)	.14
Bathroom floor	10 (71)	9 (64)	6 (38)	3 (19)	.12
Bathroom sink	6 (43)	4 (29)	0 (0)	0 (0)	1.00
***Enterococcus spp.***					
Room-Bathroom suite	17 (20)	11 (13)	19 (20)	11 (12)	.74
Patient room	5 (12)	2 (5)	6 (13)	1 (2)	.55
Bedrails	2 (14)	2 (14)	2 (13)	0 (0)	.33
Overbed table	1 (7)	0 (0)	2 (12)	0 (0)	1.00
Room sink	2 (14)	0 (0)	2 (12)	1 (6)	1.00
Patient bathroom	12 (29)	9 (21)	13 (28)	10 (21)	1.00
Toilet seat	1 (7)	0 (0)	3 (19)	4 (25)	.20
Bathroom floor	8 (57)	7 (50)	7 (44)	4 (25)	.28
Bathroom sink	3 (21)	2 (14)	3 (20)	2 (13)	1.00
**Enterobacterales**					
Room-Bathroom suite	31 (37)	25 (30)	19 (20)	20 (21)	.07
Patient room	11 (26)	12 (29)	9 (19)	5 (11)	.02
Bedrails	3 (21)	1 (7)	0 (0)	1 (7)	1.00
Overbed table	2 (14)	4 (29)	2 (12)	0 (0)	.07
Room sink	6 (43)	7 (50)	7 (44)	4 (25)	.19
Patient bathroom	20 (48)	13 (31)	10 (21)	15 (32)	.01
Toilet seat	2 (14)	1 (7)	1 (6)	4 (25)	.33
Bathroom floor	10 (71)	6 (43)	6 (38)	6 (38)	.23
Bathroom sink	8 (57)	6 (43)	3 (20)	5 (33)	.49
***C. difficile***					
Room-Bathroom suite	7 (8)	4 (5)	7 (7)	3 (3)	1.00
Patient room	2 (5)	2 (5)	3 (6)	0 (0)	.10
Bedrails	0 (0)	0 (0)	0 (0)	0 (0)	1.00
Overbed table	0 (0)	1 (7)	2 (12)	0 (0)	.33
Room sink	2 (14)	1 (7)	1 (6)	0 (0)	1.00
Patient bathroom	5 (12)	2 (5)	4 (9)	3 (6)	.52
Toilet seat	2 (14)	0 (0)	0 (0)	0 (0)	1.00
Bathroom floor	1 (7)	2 (14)	1 (6)	3 (19)	1.00
Bathroom sink	2 (14)	0 (0)	3 (20)	0 (0)	1.00

Inocula were 1.1 × 10^4^ CFU for MRSA and 5.0 × 10 ^ 3 CFU for *C. difficile*. On average, Legacy reduced MRSA by 8.6 × 10^4^ CFU (96%) compared with 1.09 × 10^4^ CFU (99%) for iQ (*p* = <.01), while Legacy reduced *C. difficile* by 1.6 × 10^2^ CFU (3%) compared with 2.0 × 10^3^ CFU (40%) for iQ (*P* = .02). Additionally, elimination of inoculated pathogens from formica surfaces was more frequent in the intervention arm overall (52% vs 81%; *P* < .01), and by disinfection cycle; 81% vs 97% (*P* = .03) for vegetative cycles and 13% vs 44% (*P* = .03) for spore cycles.

Run times were significantly shorter in the intervention arm overall (42 mins vs 22 mins; *P* < .01), and by disinfection cycle; 33 minutes vs 16 minutes (*P* < .01) for vegetative cycles and 54 minutes vs 35 minutes (*P* < .01) for spore cycles.

## Discussion

In this randomized experimental study, both the Tru-D iQ and Tru-D Legacy UV-C systems reduced environmental contamination on high-touch surfaces following patient discharge. The Tru-D iQ system generally achieved greater percent reduction at most sites, significantly so at bathroom sinks, but otherwise showed equivalent reductions. The Tru-D iQ also achieved higher elimination of inoculated pathogens across vegetative and spore cycles, suggesting enhanced performance under controlled conditions. Importantly, the iQ system completed disinfection approximately 20 minutes faster, on average.

Limitations include modest sample size, single-center design, and split-surface sampling, which may not fully represent whole-room efficacy.

In conclusion, the Tru-D iQ UV-C system demonstrated key improvements over the Legacy Tru-D Legacy system. Our findings suggest that the iQ system maintains the Legacy’s efficacy while overcoming prior limitations of bathroom coverage and cycle duration, making next-generation UV-C systems more feasible for routine adoption in acute care settings.
